# Managing crime through migration law in Australia and the United States: a comparative analysis

**DOI:** 10.1186/s40878-017-0056-0

**Published:** 2017-08-07

**Authors:** Khanh Hoang, Sudrishti Reich

**Affiliations:** Migration Law Program, ANU College of Law, Australian National University, Canberra, Australia

**Keywords:** Crimmigration, Migration, Visa cancellation, Comparative migration

## Abstract

This article examines the intertwining of migration law and criminal law — termed ‘crimmigration’ by scholars — in Australia and the United States of America, and its implications for non-citizens who engage in criminal conduct. Our comparison of the two systems demonstrates that the laws and policies in both jurisdictions are similar to a significant degree. Both have strong exclusionary policies characterised by sweeping visa cancellation/removal powers, a heavy focus on enforcement, and limited review rights. In Australia, legislative amendments in 2014 have given the executive greater powers to cancel visas and remove non-citizens on character grounds as a means of ensuring national security and public safety. This has coincided with a new law enforcement body created within the Australian Department of Immigration. These changes reflect a repurposing of migration law as a tool for managing criminal threats based on the concept of ‘risk management’. Drawing on the experience of the United States — where such a ‘risk management’ approach is entrenched — we query the utility of this shift and highlight the potential pitfalls of pursuing such a policy for Australia.

## Introduction

Paul is a 56-year-old New Zealand citizen who has lived in Australia for 36 years as a permanent resident. On 18 October 2015, he was reportedly removed from Australia and sent back to New Zealand, where he was given a voucher for a week of accommodation. He had been jailed for 13 months for ‘self-medicating’ with controlled painkillers. His removal was made possible by amendments to Australia’s migration laws in December 2014, which renders anyone who has been sentenced to a term of imprisonment of 12 months or more liable for visa cancellation and removal. Paul says he has no friends or family in New Zealand and feels that he ‘has been dumped’ by Australia (Milman, 2015).

Gabriella Portillo was 18 years old and four months pregnant at the time of her deportation from the United States to El Salvador. Portillo had fled to the United States when she was 13 on the back of a truck after being forced into sex work in El Salvador. She was abused by her family in the United States and was placed into the foster care system. She was charged with ‘arson and assault with a deadly weapon’ for which she pleaded guilty and received a 10 year suspended sentence. She agreed to pay a fine and serve inpatient treatment. Unaware that pleading guilty would trigger deportation, Portillo was deported to El Salvador, where she knows nobody except her grandmother who had initially pushed her into the sex trade (Graham, 2015).

Such stories are common-place in both jurisdictions. Paul and Gabriella are both victims of ‘crimmigration’ — the intertwining of criminal and migration laws — in their respective countries. The reliance on criminal conduct, or suspected involvement in criminal conduct, as a basis for the removal of non-citizens is one manifestation of crimmigration that has received considerable academic attention in the United States. In contrast, scholarship in the Australian context remains relatively underexplored (notable Australian works include, Foster, 2009; Grewcock, 2014, 2011, 2009; Harris-Rimmer, 2010; Nethery, 2012; Welch 2012; Van Berlo, 2015). In particular, little has been written on the subject in the Australian context since 2014 when significant changes to law and policy in this area came into effect.

This article provides a contemporary comparative analysis of this particular aspect of crimmigration in Australia and the United States. We demonstrate that United States law and policy has entrenched broad executive powers to remove non-citizens on the basis of criminal conduct. By essentially repurposing migration law as a crime management tool, the United States has sought to facilitate efficient identification and removal of ‘risky’ non-citizens as a means of ensuring national security and public safety. We use the experience of the United States as a lens through which to examine recent developments in Australian law and policy. Our comparison shows a convergence of law and policy in these two jurisdictions, and we highlight some of the potential pitfalls of this convergence for Australia.

We have chosen the United States as a comparator for Australia because the US has been a leader in its use of crimmigration laws and policies. To the extent that both states are liberal democracies that share similar legal traditions and managed migration systems, the experience of the US provides fertile ground for analysis and comparison.

Our article proceeds as follows. Part I provides an overview of the phenomenon of crimmigration and its exclusionary power in relation to non-citizens who have engaged in, or suspected to have engaged in, criminal conduct. Parts II and III outline the legal and policy frameworks governing the removal of such non-citizens in the United States and Australia, respectively. We demonstrate how, in both jurisdictions, migration laws have been repurposed as a crime management tool ostensibly to ensure national security and public safety. This repurposing is characterised by sweeping visa cancellation and removal/deportation powers, a heavy focus on enforcement, and limited rights of review or discretionary relief from removal. To the extent that there is a convergence in law and policy between the two jurisdictions, Australia appears to have transplanted a ‘risk management’ approach that has long been a feature of the United States system. In part IV, we query the normative basis of this shift and highlight the potential pitfalls of pursuing such a policy for Australia.

## I. Crimmigration and managing crime through the exclusion of non-citizens

The removal of non-citizens as an exercise of state sovereignty is a notion largely unchallenged in national and international law, subject only to limits imposed on a state by treaty and universal human rights obligations.^1^ There are many bases on which a state may remove a non-citizen from its territory, including where they have entered without permission, overstayed, or violated conditions of their visas.^2^ Scholars have observed that jurisdictions including Australia, the United States, and some in the European Union increasingly rely on criminal conduct as a basis for removal of non-citizens — including refugees and asylum seekers — from their territory (Kanstroom, 2012, 2000; Chacon, 2007; Grewcock, 2014; Stumpf, 2006; Van Berlo, 2015; Van der Woude, capital Van der Leun, & Nijland, 2014; ). This practice is one example of the increasingly blurred distinction between migration and criminal law and has resulted in a distinct legal and policy area labelled as ‘crimmigration’ (Stumpf, 2006).

Historically, criminal and migration were seen as distinct areas of law with different aims and purposes. Migration law regulates entry *at* the border whereas criminal law regulates the behaviour of citizens and residents *within* borders. Crimmigration blurs this distinction by, inter alia, attaching migration consequences — including visa cancellation, detention, removal and exclusion — for non-citizens who engage in criminal conduct *after* their entry into a country. As Aas (2014) describes, crimmigration can manifest when migration and criminal law operate under conditions of ‘interchangeability and mutual enforcement’ whereby ‘criminal law is applied not only to punish, but also to deport, while deportation is used not only for immigration purposes, but also because an individual is seen as a law and order problem (without necessarily needing to prove so with criminal law procedural means)’ (p. 525).

In many ways, the merger of these two systems is not surprising. As Stumpf (Stumpf, 2006, p. 396) — who first termed ‘crimmigration’ — describes, both migration and criminal laws are exclusionary in nature: both act as ‘gatekeepers of membership’ to society. The idea that a State is justified in preserving rights and privileges of formal citizens (i.e. members) at the expense of non-citizens (non-members) is at the core of crimmigration (Stumpf, 2006; Gibney, 2004).^3^

Reliance on criminal conduct as a basis for removal also reflects the increased securitisation of migration law through which governments seek to shield their citizens from global threats posed by non-citizens (Aas, 2011, 2014). Such threats include terrorism, drug trafficking, human trafficking and trans-national crime. Using migration laws to manage criminal threats is an attractive tool for States because it facilitates the speedy removal of unwanted non-citizens and by-passes the criminal law system, where there are greater substantive procedural safeguards (Aas, 2014; McCleod, 2012).

This merger between criminal and migration laws raises several interesting policy questions. Is it an effective way to protect national security as is claimed by politicians and lawmakers? Should we be supportive of moves to reorient or repurpose migration laws to facilitate the removal of non-citizens who engage in criminal conduct? What are some of the risks and dangers of such an approach? Our article seeks to answer these questions in relation to Australia, drawing on the experience of the United States.

## II. Crimmigration in the United States

Immigration laws in the United States have long provided the power to deport unwanted non-citizens. In 1893, the Supreme Court held in *Fong Yue Tin v United States* that the power to deport non-citizens ‘rests on the same grounds, and is as absolute and unqualified, as the right to prohibit and prevent their entrance into the country’ (Fong Yue Tin, 1893, p. 707). In the same case, it was also held that deportation is an administrative method of enforcing immigration laws and does not amount to criminal punishment (Fong Yue Tin, 1893, pp. 731–732).

Despite the existence of deportation powers, up until the late 1980s the majority of those who were deported from the United States were removed for reasons other than involvement in criminal activities (US Department of Justice: Immigration and Naturalization Service 1999, Table 60).^4^ Rather, as McLeod (2012) observed, the removal of aliens was an administrative power ‘deployed largely to advance restrictive racial, economic and ideological agendas, rather than relying upon criminal law as a foundational and independent vehicle for immigration enforcement’ (p. 117).

Liberalisation of US immigration policy during the 1950s to 1980s resulted in growing numbers of immigrants, whose presence exacerbated ongoing economic and racial tensions (McLeod, 2012). In a bid to control immigration numbers — and in particular to curb drug and contraband smuggling into the United States — legislators turned to immigration laws as a means to quickly identify and remove immigrants involved in these activities. The turning point was in 1988 when, as part of the ‘war on drugs’, the Anti-Drug Abuse Act of 1988 introduced the category of ‘aggravated felony’ as a ground for deportation, which has since been used to facilitate the removal of thousands of non-citizens from the United States.

As observed by Stumpf (2006), since the late 1980s, this aspect of crimmigration has expanded significantly in the United States through three main avenues: the expansion of classes of deportable ‘criminal aliens’; a stronger focus on enforcement and removals; and the removal of discretionary judicial relief from deportation. This has resulted in a system that grants the executive significant powers to manage crime through the identification and removal of ‘criminal aliens’ (see eg, Chacon, 2007, 2012, 2013; Kanstroom, 2000, 2012; McLeod, 2012; Stumpf, 2006).

## Expansion of classes of ‘deportable aliens’

The deportation of aliens (i.e. non-citizens) is governed by the *Immigration and Nationality Act of 1952* (INA) (contained in Title 8 of the US Code), which outlines the circumstances under which deportation can take place. Aliens may be deported for a range of offences including high speed flight from an immigration checkpoint,^5^ failure to register as a sex offender,^6^ conviction for certain firearms offences,^7^ domestic violence,^8^ and crimes of ‘moral turpitude’.^9^

As Stumpf (Stumpf, 2006, p. 383) observed, prior to 1988, the class of deportable aliens was limited to those with ‘past criminal convictions for crimes of moral turpitude, drug trafficking and some weapons offences’. The deportation of permanent residents was rare. In 1988, Congress amended the INA to include, as a deportable alien, those who had committed an ‘aggravated felony’: a wider class that included murder and firearms trafficking.^10^ Further significant amendments took place in 1996 in the form of two pieces of legislation: the Antiterrorism and Effective Death Penalty Act (AEDPA)^11^ and the Illegal Immigration Reform and Immigrant Responsibility Act (IIRIRA).^12^ These Acts further expanded the definition of ‘aggravated felony’ to include many offences that do not involve violence, including gambling, alien smuggling and passport fraud,^13^ and also reduced to one year the sentence required for a deportable ‘crime of violence’.^14^ They also applied retrospectively to cover crimes that were committed before the Acts came into force.^15^

Since then, the list of ‘aggravated felonies’ has expanded significantly to include 28 types of offences, covering a wide array of criminal conduct from minor to serious (Title 8 of the US Code (§ 1101(a)(43)). Nancy Morawetz (Morawetz, 2000, p. 1939) has described the ground as an ‘Alice-in-wonderland-like definition … as the term is defined, a crime need not be either aggravated or a felony’ under state laws. Indeed, the breadth of aggravated felonies is so wide that a single conviction for any of the listed crimes ‘invariably results in removal from the United States’ (Stumpf, 2009, p. 1723).

## Removal of discretionary relief

The 1996 amendments also removed access to judicial review that could halt deportation. From 1976 to 1996, criminal aliens had the right to petition an immigration judge to exercise a waiver of deportation under § 212(c). Judges were required to conduct hearings and could waive deportation based on factors including the length of residence in the United States, education, employment history, evidence of rehabilitation, positive contributions to the United States and evidence of good character (Ong Hing, 2006, pp. 58–60). These mitigating factors are weighed against the nature and seriousness of the crime. The waiver provisions thus allowed for some proportionality, recognising that deportation has adverse consequences not only on the individual alien, but also American citizens who are family members.

§ 304(b) of the IIRIRA repealed § 212 and replaced it with a more restrictive ‘cancellation of removal’ provision under § 240A. Under this provision, the Attorney General may cancel removal if the person: (a) has been a permanent resident for not less than 5 years; (b) has continuously resided in the United States for seven years after having been admitted under any status; and (c) has *not* been convicted of an ‘aggravated felony’. Therefore, it is the case that an alien who is convicted of an ‘aggravated felony’ is unable to obtain cancellation of removal.

While the judiciary has been hampered in its ability to prevent deportation, it has been able to place some onus on counsel to inform their clients of the consequences of pleading guilty to an aggravated felony. In the 2010 case of *Padilla v Kentucky*, a majority of the US Supreme Court (7–2) held that the right to counsel in criminal trials contained in the Sixth Amendment to the US Constitution requires, in the case where a criminal conviction leads to automatic deportation, that ‘counsel must inform her client whether his plea carries the risk of deportation’.^16^

Mr. Padilla had been a lawful permanent resident for forty years before he was caught in possession of marijuana whilst driving a truck. He pleaded guilty to drug-related charges after conferring with his counsel, who he claimed had not advised him of the consequences of deportation. Before the Supreme Court, he claimed that he would not have gone to trial and pled guilty, but for receiving incorrect legal advice. Crucial to the majority’s finding was that, since deportation virtually flowed automatically from a criminal conviction, it was an inextricable part of the criminal process to which counsel must alert his or her client. The Court recognised that:

*‘The landscape of federal immigration law has changed dramatically over the last 90 years. While once there was only a narrow class of deportable offenses and judges wielded broad discretionary authority to prevent deportation, immigration reforms over time have expanded the class of deportable offenses and limited the authority of judges to alleviate the harsh consequences of deportation. The “drastic measure” of deportation or removal, . . . is now virtually inevitable for a vast number of noncitizens convicted of crimes’.* 17

Commentators have noted that the judgment in Padilla is important in that it may help prevent wrongful deportations (Kanstroom, 2012, 2011; Vazquez, 2011). However, the judgment is unlikely to help those non-citizens who cannot afford legal representation or who are unable to negotiate the criminal law system and plea bargain in a manner that would minimise their risk of deportation. As Vazquez (2011: p. 179) highlights, for these non-citizens, ‘salvation typically depend[s] on whether his court appointed attorney or public defender has the ability and knowledge necessary to negotiate a plea bargain during the criminal proceedings that will result in a disposition that will prevent the noncitizen defendant from being deported’.

## Increased focus on law enforcement and removal

The expansion of the grounds for deportation and the removal of discretionary waivers were coupled with an increased focus on law enforcement, such that ‘immigration enforcement has come to parallel criminal law enforcement’ (Stumpf, 2006, p. 386). This can be seen in the transfer of immigration control from the Department of Justice to the Department of Homeland Security (DHS) in 2002, and the creation of two immigration enforcement agencies — Immigration and Customs Enforcement (ICE)^18^ and the U.S. Customs and Border Protection^19^ — whose responsibilities for enforcing immigration laws are largely indistinguishable from a criminal law enforcement organisation (Stumpf, 2006: p. 387). As Chacon (2012) highlights, the budgets of both DHS and ICE grew substantially in the 2000s and there was an increased focused on prosecuting immigration laws and crimes (pp. 630–640).

An example of increased law enforcement efforts can be seen in cooperative schemes between Federal and State law enforcement agencies. The 1996 amendments allowed non-federal law enforcement agencies to enter into agreement with the government to allow them to enforce federal immigration laws.^20^ This facilitated programs such as the 287(g) program, under which the DHS could deputise state and local sheriffs to enforce immigration laws (8 U.S.C. § 1357 (g) (2000)). Some scholars suggest that this form of ‘shadow enforcement’ has incentivised state police officers to disproportionately target immigration violations and immigrants rather than focus on criminal law enforcement, partly because federal reimbursement is available to states under the schemes (Mcleod, 2012, p. 146; Sweeney, 2014). Capps, Rosenblum, Rodriguez, and Chisti (2011) found that under the 287(g) program, some jurisdictions took a universal enforcement approach by apprehending any immigrant who came in contact with the criminal system, while others more narrowly targeted national security threats. This suggests haphazard rather than uniform and consistent efforts to identify and remove the most hardened criminals.

Other programs, such as the Secure Communities Program — which ran from 2008 to 2014 and covered 3181 jurisdictions in 40 states — mandated that whenever a state or local jurisdiction submits a person’s fingerprints to the FBI, it is also checked against DHS’s database for immigration violations (Kubrin, 2014). A fingerprint match prompts ICE officers to investigate and act on a person’s immigration status, which also facilitates speedy removals. However, commentators have questioned whether the program has actually resulted in a safer community, as the program seems to have caught mainly non-citizens who committed low level crimes rather than hardened criminals (Kubrin, 2014; Treyger, Chaflin, & Loeffler, 2014).

The Secure Communities Program was discontinued in late 2014 and replaced with the Priority Enforcement Program (PEP) (U.S. Immigration and Customs Enforcement, 2016). Under the PEP, when an individual is arrested by local law enforcement or when fingerprints are submitted to the FBI, biometric information is also sent to ICE to determine whether the individual should be removed as a matter of priority under policy. In October 2014, the DHS updated its policy guidance for the apprehension, detention and removal of aliens. The overriding emphasis was that enforcement and removal policies should ‘continue to prioritise threats to national security, public safety and border security’. (U.S. Department of Homeland Security, 2014). The policy outlines three levels of priority. It is of note that priority 1 — the highest category — includes aliens convicted of an ‘aggravated felony’ as defined in the INA at the time of conviction. This policy guidance thus continues to provide a clear mandate for ICE to aggressively pursue the removal of ‘aggravated felons’.

The heavy focus on enforcement, aided in part by the wide definition of an ‘aggravated felony’, has resulted in large numbers of aliens being removed from the United States. For the period between 1908 and 1980, approximately 56,000 aliens were deported based on previous criminal convictions. In contrast, for the fiscal year 2015 alone, ICE removed 235,413 individuals of whom 59% (139,368) were said to have previously been convicted of a crime. (US Immigration and Customs Enforcement, 2015).

## III. Australia’s approach: following in the footsteps of the United States

In this section, we trace the rise of ‘crimmigration’ in Australia with particular focus on the legislative and policy changes in 2014 and 2015. These changes expanded the executive’s already sweeping powers to cancel a person’s visa on ‘character grounds’. The expansion of cancellation powers coincided with the creation of the Australian Border Force (ABF), a new law enforcement body within the Department of Immigration focused on compliance and enforcement.

It will be readily apparent that, from a crimmigration perspective, parallels to the United States system are striking.

Like the United States, immigration laws in Australia have long provided the executive with power to exclude non-citizens from Australia (Crock & Berg, 2011; Grewcock, 2014). Under the Australian Constitution, the legislature has the power to make laws with respect to ‘aliens’ and ‘immigration and emigration’. The courts have held that the reach of the ‘aliens’ power is such that a person who is not a citizen is an ‘alien’ for the purposes of constitutional and migration law.^21^ Non-citizens — irrespective of how long they have been in Australia or the fact that they are permanent residents — are therefore susceptible to removal from Australia under migration laws.

The entry and stay of non-citizens in Australia is governed by the *Migration Act,* 1958 (Cth) (the Act). Section 4 provides that the objective of the Act is to ‘regulate, in the national interest, the coming into, and presence in, Australia of non-citizens’. To advance this object, the Act provides for visas as the only source of lawful presence (Migration Act, 1958: ss 14, 15) and provides for the removal of non-citizens whose presence in Australia is not permitted by the Act (that is, unlawful non-citizens) (s 198). A visa holder whose visa is cancelled becomes an ‘unlawful non-citizen’ and is liable for detention and removal from the country.

There are two powers in the Act under which a non-citizen can be deported or removed from Australia on criminal or character grounds. A non-citizen may be deported by order of the Minister for Immigration (the Minister) under section 200 of the Act for having been convicted of a criminal offence for which they were sentenced to imprisonment for at least one year. A second power is found under section 501, whereby a non-citizen can have his or her visa refused or cancelled if the person does not satisfy the the Minister that he or she passes the ‘character test’, as defined in subsection 501(6).

The section 200 criminal deportation power has always recognised the special position of long-term residents. Section 12 of the original Act prevented someone from being deported if the offence was committed after they had been residing in Australia for 5 years. In 1983 this exemption was extended (*Migration Amendment Act 1983* (Cth) s10), such that after 10 years of residence as a permanent resident a non-citizen was protected from deportation (Migration Act, s201) (Foster, 2009). Until the early 1990s, it was the deportation power that was used to remove criminal non-citizens from Australia.

A character-based visa cancellation power was first introduced into the Act in 1992 (as section 180A). It gave the Minister power to refuse a visa to, and cancel the visa of, a non-citizen who the Minister was satisfied was not of good character. This first ‘character test’ was quite rudimentary and gave considerable discretion to the Minister. Unlike the criminal deportation power, there was no exemption for long-term permanent residents. However, as Foster (2009) has documented, the original rationale for the character regime was to give the Minister the power to *exclude* undesirable entrants (by refusing a visa) rather than remove those who were residing in Australia. That is, the character cancellation provision was not intended to supplant or circumvent the criminal deportation power.

Nevertheless, over time, the executive made a shift in its administration of the two powers: increasingly the section 501 character cancellation power was used to achieve the removal of non-citizens deemed to fail the character test, and conversely, the deportation power was relied on less and less. The criminal deportation power, with its inbuilt exemption for permanent residents of more than 10 years residency, is now very rarely used.

Emblematic of this development were amendments made to the character cancellation power and the character test in 1998 (*Migration Legislation Amendment (Strengthening of Provisions Relating to Character and Conduct) Act* 1998 (Cth)). These amendments strengthened the Minister’s powers to cancel visas and remove non-citizens who did not pass the ‘character test’, as defined in subsection 501(6). The amendments introduced additional grounds under which a person is deemed to fail the character test. These included where a person has been sentenced to death or life imprisonment, has had an association with persons or organisations involved in criminal conduct, or where there is a significant risk that the person will engage in criminal conduct in the future. One of the significant additions was that a person with a ‘substantial criminal record’ did not pass the character test. A substantial criminal record was defined to include a sentence of imprisonment for 12 months or more; or two or more sentences that total 2 years or more. The 1998 amendments also introduced provisions allowing the Minister to cancel a visa without applying the rules of natural justice (such as prior notification and the right to be heard) and giving the Minister power to overturn a (more favourable) decision of a merits review tribunal.

### Expanding the grounds and procedures for character cancellation

In 2014, further significant amendments were made to the visa cancellation regime by the *Migration Amendment (Character and General Visa Cancellation) Act 2014* (Cth) *(Character Act).* The effect was a broadening of the classes of persons that are liable for character cancellation, further reducing avenues for merits review, and increased executive power to overturn decisions of the merits review bodies.

In the paragraphs below, we highlight the changes to the character test resulting from the *Character Act*. It is important to note that some of these grounds do not require any criminal offence to have been committed, let alone a criminal conviction. The threshold for some is simply the ‘reasonable suspicion’ of the Minister or alleged involvement in criminal activity. While some may suggest that this is not a crimmigration development — since no criminal conviction is necessary for cancellation — we say that it is, because it provides an avenue for removal of persons alleged to have engaged in criminal activity, but in a manner that bypasses the rigours of the criminal justice system. In effect, such provisions represent over-criminalising non-citizens by effectively “criminalising” actions that would not constitute crimes in the criminal law context.

### Substantial criminal record and mandatory visa cancellation

The *Character Act* amended the definition of ‘substantial criminal record’ to include where a person has been sentenced to a series of lesser terms of imprisonment that add up to 12 months or more (previously 24 months). The government’s rationale was that a series of sentences such as these ‘raise serious concerns about a person’s character, including that there may be a high risk of recidivism and a clear disregard for the law’ (Explanatory Memorandum, 2014, 12). Aggregating sentences in this way significantly lowers the threshold and broadens the cohort of non-citizens liable for visa cancellation.

The *Character Act* also introduced for the first time in Australia a mandatory character cancellation power. Under s 501(3A), the Minister *must* cancel a visa, without notice to the visa holder, if the person:

- has a ‘substantial criminal record’, having been sentenced to death, life imprisonment or a term of imprisonment of 12 months or more; or

- has been convicted or found guilty of a sexual offence involving a child; and

- is serving a sentence in prison for an offence against a law of the Commonwealth, a State or a Territory.

The effect of this provision is that non-citizen prisoners caught by the provision will have their visas mandatorily cancelled, usually prior to their release date. After having served their term of imprisonment, the non-citizen would then be placed in immigration detention and subject to removal – which can be delayed for months. The rules of natural justice do not apply to decisions made under this provision. Hence a visa holder will not be given prior notice that their visa is being considered for cancellation and will not be given an opportunity to respond. The affected non-citizen has the right to seek revocation of the mandatory cancellation decision under section 501CA, but only within a narrow 28-day window. However, the Minister retains the ultimate power to overturn any decision to revoke the cancellation made by a delegate or the Administrative Appeals Tribunal (a merits review body). This power is exercisable by the Minister only and the Minister must be satisfied that it is in the ‘national interest’ (s 501BA).

### Membership of an organisation or group involved in criminal conduct

The *Character Act* also lowered the threshold for the criminal association ground for cancellation from actual association to making it sufficient for the Minister to ‘reasonably suspect’ that ‘a person has been or is a member of or has or had an association with, a group, organisation or person ... and that the group, organisation or person has been or is involved in criminal conduct’ (s 501(6)(b)(i)). The Explanatory Memorandum makes it clear that ‘mere membership’ of such organisation or group is sufficient to fail the character test. There is no requirement that there be a ‘demonstration of special knowledge of, or participation in, the suspected criminal conduct by the visa applicant or visa holder’ (Explanatory Memorandum, 2014, 9). Indeed, in *Roach v Minister for Immigration and Border Protection* [2016] FCA 750, Perry J concluded that this membership provision, as amended by the *Character Act*, is in effect a deeming provision. That is, a person who is suspected of being a member of a group or organisation that is suspected of being involved in criminal activities will automatically fail the character test (similarly to someone who has a ‘substantial criminal record’). [cited with approval by the Full Court of the Federal Court in *Taulahi v Minister for Immigration and Border Protection* [2016] FCAFC 177].

Further, the Act provides that a person does not pass the character test if the Minister *reasonably suspects* that the person has been, or is, involved in a range of other serious offences. These include offences related to people smuggling, trafficking in persons, crimes of genocide, crimes against humanity, war crimes, crimes involving torture or slavery or a crime that is otherwise of serious international concern (Migration Act, s 501(6)(ba)(i)-(iii)). There is no requirement that the person has been convicted of the offence.

### Future conduct on the basis of ‘risk’

The *Character Act* also lowered the bar for cancelling a visa on the basis of potential future conduct. A person does not pass the character test if there is a ‘risk’ (previously, a *significant* risk) that the person would engage in criminal conduct, or harass, molest, intimidate or stalk a person, or vilify a segment of the community, or incite discord or in any way represent a danger to the Australian community (Migration Act, s 501(6)(d)). The intention of the provision is that the level of risk ‘is more than a minimal or trivial likelihood of risk, without requiring the decision-maker to prove that it amounts to a significant risk’ (Explanatory Memorandum, 2014, 11). Again, no offence needs to have been committed and no conviction is necessary to trigger the cancellation.

It can be seen that the *Character Act* has significantly widened the cohort of non-citizens who are liable for visa cancellation and removal.

### Discretionary exercise of the cancellation power

Once a ground for character visa cancellation referred to above is enlivened, either the Minister (exercising power personally under s 501(3)) or a delegate of the Minister (under s 501(2)), can cancel a visa. There are two major differences between these powers. If the decision to cancel is made by a delegate of the Minister, the decision is subject to the ‘rules of natural justice’ and the code of procedure set out in the Act (Migration Act, s 501(5)). These require that the person be notified of the intention to cancel and given a chance to respond and make arguments as to why the visa should not be cancelled (Migration Act, s 51A—64). The decision of a delegate is reviewable by a merits review body, the Administrative Appeals Tribunal (Migration Act, s 500(1)(b)).

In contrast, where the Minister acting personally exercises the power to cancel, that power is exercised without regard to ‘natural justice’ and no merits review is available (*Migration Act,* ss 501(3) and 500). This power can only be exercised where the Minister considers that the cancellation is in the ‘national interest’; a term not defined in the legislation.

A second difference between the delegable and the personal powers lies in how the cancellation power is exercised. Where the decision is made by a delegate, the decision-maker must have regard to binding directions issued by the Minister under s 499 of the *Migration Act*. These set out factors that a delegated decision-maker must take into consideration when exercising the discretion to cancel. A number of these directions have been issued since 1992 in relation to character cancellation decisions, and require decision-makers to weigh up relevant factors including the protection of the Australian community, the likelihood of recidivism, general deterrence, the best interests of any child, the person’s links and associations with Australia and relevant international obligations (the latest is Ministerial Direction No 65, 2014) (Morrison, 2014). In contrast, where the Immigration Minister decides to make the decision to cancel personally in the ‘national interest’, the Minister is not required to take into consideration any of the factors under the s 499 direction.

### Limited avenues for judicial intervention

Given the increased powers given to the executive to cancel visas on ‘character’ grounds, it is reasonable to consider the extent to which the judiciary can provide a check on the exercise of such powers. We suggest that, to the extent that the powers rest on the subjective assessment of the Minister or decision-maker exercising the discretion to cancel, there is little scope for the judiciary to intervene.

The starting point is that decisions made by the Minister (and those of his or her delegates) to cancel a visa on character grounds are open to judicial review. However, the judiciary can only review the legality of the decision – whether it was made within the power conferred by the legislation and within Constitutional bounds, or whether it is void for jurisdictional error.^22^ The court cannot revisit the facts of the case, does not have power to consider mitigating and other factors, and cannot conduct its own balancing of the countervailing considerations and arrive at a different outcome.

Since the passing of the Character Act, the courts have been called upon to review numerous visa cancellation decisions made under s 501. Many of these cases have involved long-term permanent residents where the consequences of visa cancellation and removal from the country are particularly harsh.

One ground for seeking judicial review is that the decision of the Minister or delegate was so unreasonable or disproportionate that it is beyond power. The case of *Minister for Immigration and Border Protection v Stretton* [2016] 237 FCR 1 is representative of legal challenges to character cancellation decisions on the grounds of reasonableness. Mr. Stretton migrated to Australia with his family in 1961 at the age of 6 years and had lived in Australia for more than 50 years when his visa was cancelled. The Minister cancelled his visa, using his personal discretionary power under s 501(2) following Mr. Stretton’s conviction for the sexual abuse of his granddaughter.

Mr. Stretton argued in the Federal Court (*Stretton v Minister for Immigration and Border Protection (No 2)* [2015] FCA 559) that the decision to cancel his visa was unreasonable given the harshness of the consequences for the applicant as against the acknowledged low risk of him reoffending. The Federal Court quashed the Minister’s decision finding that the decision was unreasonable and amounted to ‘using a sledgehammer to crack a nut’. However, on appeal by the Minister, the Full Court of the Federal Court reinstated the Minister’s decision. The court acknowledged the particular difficulties inherent in cases of long term residents:

*The exercise of that power in relation to a non-citizen who has been in this country for many years, with strong and deep social, family and human roots here is bound to be complex and difficult. There can be no doubt that one aspect of the scope and purpose of s 501 is the protection of the Australian community, including here vulnerable young children. The decision to remove Mr. Stretton from Australia will cause hardship to him, and his family, in particular the breaking of family relationships of many years; further, the removal of someone from Australia who has spent much of his life here (arriving as a child of six years) itself has a quality of harshness that might, in other statutory contexts, together with the effect on him and his family, bespeak unjustness, arbitrariness or disproportion of response. Whilst not a citizen of Australia, Mr. Stretton has lived here since he was a small boy. His human frailties are of someone who has lived his life here, as part of the Australian community* [para 15].

The court found that the s 501 cancellation power gives the Minister a ‘genuinely free discretion’ in the exercise of which ‘reasonable minds may differ’. It noted that it is critical that the courts ‘in exercising a judicial review function … not exceed their supervisory role by undertaking a review of the merits of an exercise of discretionary power’ (at [56] citing *Minister for Immigration and Citizenship v Li* [2013] HCA 18; 249 CLR 332). As expressed by Wigney J (para 92) ‘in circumstances where reasonable minds might differ about the outcome of, or justification for, the exercise of power, or where the outcome falls within the range of legally and factually justifiable outcomes, the exercise of power is not legally unreasonable simply because the Court disagrees, even emphatically, with the outcome or justification.’

The courts have also taken a similar approach to the concept of ‘national interest’ necessary for the exercise of the Minister’s personal powers to cancel a visa. In *Gbojueh v Minister for Immigration and Citizenship* (2012) *202 FCR 417,* the Federal Court of Australia held at [44] that the ‘national interest’ calls for a ‘broad evaluative judgement’ and that ‘the minister is left largely unrestrained to determine for him or herself what factors are to be regarded as relevant when determining whether the cancellation or refusal of a visa is in the national interest’. Kinslor and English (2015) have argued that, beyond the necessity to act rationally, ‘the only limitation arising from the case law in relation to the national interest requirement is a requirement to consider potential harm to the Australian community’ (p. 47). In practice, decisions to cancel by the Minister are often made for political reasons, as politicians do not want to be perceived as being ‘soft’ on migrants who have committed crimes (Crock & Berg, 2011, pp. 519–520). Decisions to cancel visas may also be influenced by media coverage, which is generally hostile to prisoners affected by s 501 (Grewcock, 2011).

Thus, to the extent that most cancellation decisions involve an exercise of discretion based on ‘reasonable suspicion’, an assessment of ‘risk’ or ‘national interest’, it appears that only the most egregious of decisions would be overturned on judicial review.

### Shifting focus on law enforcement

The *Character Act* changes coincided with the quasi-militarisation of Australia’s immigration Department and a changing culture focused on enforcement. On 1 July 2015, the functions of the Department of Immigration and Border Protection and the Australian Customs and Border Protection Services were merged into a new operational entity: the Australian Border Force (the ABF). The ABF is described as a disciplined, uniformed, front-line agency with significant service and enforcement functions (Australian Border Force, 2016).

Armed with a new focus on enforcement, the Department of Immigration and Border Protection lost no time in exercising its expanded powers for cancelling visas and removing non-citizens. In the Department’s annual report for the financial year 2014–2015, it claimed as a ‘major achievement’ that ‘[w]ith the commencement of the Migration Amendment (Character and General Visa Cancellation) Act 2014 on 12 December 2014, the Department has been able to cancel visas on the basis that the visa holder presents or may present, or would or might present a risk to the health, safety or good order of the Australian community.’ (Department of Immigration and Border Protection, 2015, 155). In the financial year 2014–2015, 580 character cancellations decisions were made, and in 2015–2016, 983 cancellations were made (Department of Immigration and Border Protection, 2016). This represented more than a tenfold increase on the year immediately before the Character Act took effect (only 76 cancellations).

## IV. Comparative analysis: managing crime through migration

The above demonstrates that the laws and policies in Australia and the US governing removal of non-citizens on the basis of criminal conduct are now remarkably similar, with a few exceptions.

In terms of the legal grounds for removal, both have extensive grounds on which a non-citizen can be removed for involvement in criminal conduct. It is arguable that the grounds in Australia are more expansive than the US, as many do not require a conviction or, in some instances, even the commission of a crime: mere suspicion of or alleged involvement in criminal conduct is enough to trigger visa cancellation and removal.

The Australian system has greater scope for consideration of individual circumstances and mitigating factors, since most of the cancellation decisions require an exercise of discretion by the decision-maker. In particular, where the decision to cancel is made by a delegate of the Minister, the decision-maker must take into account mitigating circumstances of the individual against any risk that the person may pose to the Australian community. Even so, it is by no means clear that mitigating factors have been applied in a consistent and fair manner in exercising the discretion to cancel in the past (Nethery, 2012). Further, while there appears to be *some* scope for the judiciary to review cancellation decisions in Australia, these avenues are limited due to the wide scope of the discretionary powers. In contrast, in the United States, once a person has been convicted of an ‘aggravated felony’, there is no room for mitigating factors against the deportation to be considered by the judiciary. While the decision in *Padilla v Kentucky* provides a measure of protection, it provides little relief to those who cannot access legal assistance.

Both jurisdictions have immigration departments that are focused on compliance and enforcement aimed at the protection of the country’s borders and national security. The creation of the Australian Border Force invites comparison to the DHS and ICE, and represents a marked shift in Australia’s migration policy, as the immigration department has not traditionally been focused on compliance and enforcement but rather on harnessing the economic and social benefits of migration for Australia in the context of a managed migration program.

In the rest of this section, we focus our analysis on the implications of Australia’s new laws and policies. It is clear that the laws are intended to facilitate the removal of individuals who are deemed a ‘risk’ to the Australian public. Drawing on the experience of the United States — where this risk management approach has been entrenched — we query the normative basis of this shift in law and policy and point to some potential pitfalls.

### National security and the protection of the community: the utility of a ‘risk management’ approach

The Australian government views the *Character Act* and the ABF as necessary to ensure national security and the protection of the Australian public from dangerous non-citizens. These changes were made in recognition that threats from ‘terrorists and violent extremists is both real and growing’ (Department of Immigration and Border Protection and Australian Customs and Border Protection, 2014, p. 12). As the Explanatory Memorandum (2014, Attachment A) for the Character Act emphasised, the amendments are aimed at ‘protecting the Australian community from *the risk of harm* by non-citizens’. In its 2014–2015 annual report, the Department cited the amendments to the cancellation framework as part of its ‘*risk-based* response to non-citizens who have breached immigration law or pose a character or national security risk’ (Department of Immigration and Border Protection, 2015, 159). Immigration Minister Peter Dutton, in a media release concerning the massive increase in character visa cancellations since the legislative amendments were passed, emphasised that such actions ‘prove the Government is serious about protecting Australians from foreign criminals’ (Dutton, 2015).

The Department’s 2015 annual report makes clear that the high levels of visa cancellation and the legislative amendments that empower them ‘reflect the Government’s position that travelling to, and remaining in, Australia is a privilege, not a right, and that any non-citizen who would seek to do us harm or who chooses to breach the law or who fails to uphold the behavioural standards expected by the Australian community should expect to be refused entry or have their visa cancelled.’ (Department of Immigration and Border Protection, 2015, p. 59). When referring to those whose visas had been cancelled, the Immigration Minister stated: ‘frankly they’re detracting from the Australian society, not adding to it. They should be removed from our shores as quickly as possible.’ (Gribbin, 2015).

Such statements reveal a number of underlying notions and assumptions: that non-citizens who commit crimes are *prima facie* a risk to security; that non-citizens (regardless of the length of their residency) have no right to remain in Australia; that staying in Australia is a privilege; that those who are not citizens are ‘foreigners’, regardless of how long they have lived in Australia or their ties with the Australian community; and that the risk posed by non-citizens can be effectively managed by the Government. Whilst noting that the new cancellation powers would result in separation of the family unit, the Minister explained that the amendments were necessary to ‘enhance the Act’s powers in the interest of national security and maintaining public order and safety, by strengthening my department’s ability to identify, assess and reduce *any risk* to the Australian community that a non-citizen may present’ (Explanatory Memorandum, 2014, Attachment A).

In our view, these developments indicate a significant acceleration in the merger of migration and criminal laws. It is clear that the new laws are predicated on a ‘risk management’ approach that seeks to ensure national security and public safety by efficiently identifying and removing cohorts of non-citizens deemed risky. As we suggest below, there are reasons to be cautious about the utility of a risk management approach. A removal regime based on subjective individual risk assessments is likely to result in over-criminalization, and can be used in a manner that disproportionately affects long-term residents.

### The United States experience: a cautionary tale

In examining the utility of a risk management approach, we suggest that the experience of the United States is instructive for Australia. In the United States, the evidence is far from clear that using migration law as a crime management tool — with a particular emphasis on risk identification — has enhanced ‘national security’ or public safety. Even if it has, the laws have facilitated the removal of non-citizens for relatively minor crimes, with devastating effects on US citizens and families (Kanstroom, 2012).

Teresa Miller (2003, 2005) argues that in the post September 11 era the United States has used migration law to govern crime and reduce risks to public safety.^23^ Drawing upon Feeley and Simon’s (1992, p. 452) ‘new penology’ she highlights that the US system ‘subjects a broad spectrum of non-citizens to harsh immigration consequences that are often only indirectly related to terrorist conduct’ (Miller, 2005, p. 101). The risk management approach incentivises enforcement bodies to achieve punitive outcomes in order to ‘argue that the United States is more secure simply because more members of a population perceived as threatening have been apprehended, detained and deported’ (Miller, 2005, p. 123). Or, as Stumpf (2006, p. 413) puts it, using migration law as a means to control crime is necessary because it is ‘infeasible to acknowledge the state’s ability to control crime is limited’. The executive must be seen to be doing *something* to mitigate border security threats. Indeed, scholars have highlighted that, by linking migration and crime control at the level of political and social discourse, States have been able to justify legislative changes that fit within the crimmigration framework (Sklansky, 2012; Van Berlo, 2015; Van der Woude et al., 2014).

Chacon (2012) argues that crimmigration in the United States can be characterised as ‘over-criminalizing’: creating too many crimes and criminalising things that should not be crimes. Chacon (2013, p. 90) has argued in relation to the 1996 amendments and those after September 11 that:

*The breadth of the resulting provisions raises serious questions as to whether the law is tailored to address an appropriately narrow class of security threats. Paradoxically, despite the significant expansion of these categories, the number of immigrants removed on security and terrorism grounds has contracted, not expanded, over the course of the past decade.*

Indeed, the statistics from DHS reveal that very few non-citizens have been removed from the United States for terrorism related offences. For example, a comparison of deportation proceedings initiated in immigration courts in the decade before and after 9/11 reveal that proceedings on national security and terrorism grounds had decreased, while the number of removals for immigration based grounds had increased (TRAC Migration Report, 2011). During the Obama administration, it has been reported that as many as two-thirds of the two million non-citizens deported had ‘committed minor infractions, including traffic violations, or had no criminal record at all’ (Thompson & Cohen, 2014).

Thus, as Chacon (2007) has observed, while the laws are touted as necessary to ensure ‘national security’ in the United States, in practice they appear to be aimed at basic crime control. She argues that this conflation between national security and personal security has done little to enhance national security. Non-citizens are rarely removed on ‘national security’ grounds, yet the US Government characterises non-citizens as being a security risk in order to justify harsh immigration laws. As a result, immigration law has been repurposed as a powerful ‘adjunct to the criminal justice system, but one that lacks comparable judicial oversight, and operates to facilitate arbitrary and excessive forms of punishment’ (Chacon, 2013, p. 93).

Thus, to the extent that recent developments in Australia represent a similar repurposing of migration law as an adjunct to the criminal justice system, we suggest that it carries inherent risks. In particular, the recent legislative and policy changes allow decision-makers to exercise discretion in a punitive manner, with considerable cost to individuals and their families.

For example, while the intended targets of the 2014 Australian amendments were identified as hardened or at least serious offenders — ‘those who have committed sexual assault, are 'gangsters', bikie gang members, engaged in physical assault or murder 'or something like that' (Medhora, 2015, quoting former Immigration Minister Scott Morrison) — it appears that lesser criminals, who pose little threat to national security, are also getting caught by the legislation. In his report after a country visit to Australia in 2016, the UN Special Rapporteur on the human rights of migrants, François Crépeau, was critical of the new cancellation regime. In relation to s 501, he noted that ‘the lack of clarity of the provisions could also risk a politicized and biased use of controls, and be in violation of the principle of legality’, and that the powers given to the Minister ‘does not give the appropriate level of oversight to the country’s judiciary’ (Crépeau 2017, p. 10). Further, the report stated that:

*The Special Rapporteur met with detainees who had had their visa cancelled, revoked or not renewed because of minor offences, committed sometimes many years previously, such as traffic violations or misdemeanours. This legislation has resulted in detainees being treated as if they had committed serious crimes.*

In December 2016, the Commonwealth Ombudsman, Colin Neave, issued a report into the administration of s 501 by the Department of Immigration and Border Protection since the new laws were enacted in 2014. Statistics given to the Ombudsman by the Department showed that 1219 visas had been cancelled between 1 January 2014 and 29 February 2016. Of these, less than 10 were for reasons of national security or organised crime, while the four highest categories were other violent offence (214), assault (210) and drug offences (148) and other non-violent offence (111) (Neave, 2016, pp. 6–7).

While it may take a number of years for solid trends and evidence to emerge, it is concerning that the numbers of visas cancelled have risen dramatically since 2014 and that many have had visas cancelled for minor offences.

### Impact on long-term residents: the need for proportionality

We predict that repurposing migration laws as a tool of crime control coupled with a focus on enforcement will inevitably lead to greater numbers of removals. In the United States, removal numbers are touted as the hallmark of the government’s success in protecting the community. However, such numbers by themselves paint a limited picture. A closer examination reveals that the haste in which the government seeks to remove non-citizens has a punitive effect, particularly on long-term permanent residents.

Statistics from the US show that between 1997 and 2006, ‘aggravated felons’ who had been removed from the United States had been in the country an average of 15 years. (TRAC Immigration, 2006). In addition, during the period between 1989 and 1995, judges on the Board of Immigration Appeals had waived 51% of deportation cases (Chacon, 2007, p. 1845). This suggests that when one is allowed to consider a wide range of mitigating factors — including the time spent in the United States and the strength of family ties — there are often good reasons to waive deportation.

As Stumpf (2011) argues, the United States’ approach wrongly ‘narrows the decision whether to shut the noncitizen out of the national community to a single moment in time: the moment of the crime that triggers the potential for deportation’ (p. 1710). It does not adequately consider any factors or events either before or after the commission of the offence. In this way, crimmigration turns the membership hierarchy on its head, because the laws mandate the removal of a permanent resident who has been in the country for many years in the same manner as a tourist who has committed a crime (Stumpf, 2011, p. 1740). Further, whereas a citizen is afforded the protection of the criminal law justice system when they commit a crime, a non-citizen who commits *the same crime* is susceptible to removal notwithstanding any other connections with the country.

Removal necessarily affects long-term permanent residents more than it affects temporary visa holders, due to familial, cultural and other bonds the person has built with the country over time. The devastating impact of removal laws on US families, particularly children, is well documented (Kanstroom, 2012; Lonegan, 2007). United States scholars have — in our view rightly — called for a more proportionate approach: one that allows for principled discretion balancing the utility of removal with the rights and interests of non-citizens coupled with adequate judicial oversight (Kanstroom, 2012; McLeod, 2012).

### Lessons for Australia

The fact that the enforcement and deportation laws are aimed at long-term permanent residents as much as temporary residents has been made explicit by the former Minister for Immigration, Scott Morrison (2015):

*[A]t the end of the day, if you’re here on a visa and you’ve committed sexual assault, if you’re a gangster, if you’re a bikie gang member, if you’ve engaged in physical assault or murder or something like that, you’ve worn out your welcome in this country.* ***I don’t care how long you’ve been here. You’ve worn out your welcome if you’re here on a visa.*** *[emphasis added].*

The political inference is that the government should be trusted to exercise discretion to remove non-citizens who pose a serious threat to Australia. Yet, despite the drastic consequences attached to removal, the system provides few checks and balances to the exercise of discretion. As noted above, decisions of the Minister are not subject to procedural fairness obligations. While judicial review of cancellation decisions is possible, the grounds for review are limited. The result is that the Minister has considerable personal power to decide which non-citizens are a risk to national security and public safety and cancellation can be made without considering mitigating circumstances. Even when decisions are made by a delegate, Ministerial Direction 65 requires the decision-maker to have regard — as a primary consideration— to the safety of the Australian public and the risk of the person offending or reoffending. In contrast, the impact on the offender, the strength, nature and duration of their ties to Australia are only listed as ‘other considerations’.

Coyle and Keyzer (2016) argue that the cancellation regime’s focus on risk assessment is ‘akin to using a sledgehammer to crack a nut’, drawing on scientific research that suggests that risk models have poor predictive capacity when applied to individuals (p. 88). If that is correct, giving greater subjective powers to decision-makers in this context is concerning. As Nethery (2012) notes, the ‘mechanical’ nature of decision-making according to specific and inflexible criteria within the Department means that cancellations are made ‘without the checks and balances usually associated with administrative decisions’ (p. 735). The introduction of subjective risk assessment may, in the current framework, make it more palatable for decision-makers to cancel visas. That is, once a person is identified as a being *a risk* — no matter how big or small — to public security, the default position is to cancel their visa without proper consideration of other mitigating factors.

All of this is concerning because many of the people removed or detained awaiting removal include long-term residents, including those who arrived in Australia as children and have spent the greater part of their lives living in Australia as part of the Australian community. These people know no other home, having left their birth country as minors. They are embedded in the Australian community – work, pay taxes, have Australian partners and Australian born children. For all intents and purposes they are Australian – certainly they are the product of Australia, having been raised, educated and socialised in Australia. The case of Stefan Nystrom has become a rather notorious example of this (Foster, 2009). Mr. Nystrom migrated to Australia as an infant of 25 days and lived the next 33 years in Australia. Based on his criminal record, he was deported back to Sweden where he had no relatives and did not speak the language. The Federal Court noted in *Nystrom v Minister for Immigration and Multicultural and Indigenous Affairs* [2005] FCAFC 121:

*It is one thing to say that the responsibility to determine who should be allowed to enter or to remain in Australia in the interests of the Australian community ultimately lies with the discretion of the responsible minister. That has little to do with the permanent banishment of an absorbed member of the Australian community with no relevant ties elsewhere. The appellant has indeed behaved badly, but no worse than many of his age who have also lived as members of the Australian community all their lives but who happen to be citizens. The difference is the barest of technicalities. It is the chance result of an accident of birth, the inaction of the appellant’s parents and some contestable High Court decisions. Apart from the dire punishment of the individual involved, it presumes that Australia can export its problems elsewhere.* 24

While it may take some years for trends to emerge, there is potential that cases like Nystrom will become increasingly normalised and that long-term permanent residents will be disproportionately affected.

## Conclusion

This paper has considered a particular aspect of crimmigration in Australia and the United States: the removal of non-citizens on the basis of involvement in criminal conduct. It has demonstrated that both jurisdictions exhibit similar laws and policies to manage criminal risks associated with non-citizens. Both jurisdictions have expansive grounds upon which a non-citizen can be removed for involvement in criminal and related conduct, a strong focus on quick and efficient removals, and limited avenues for judicial review.

For Australia, the accelerated merger between criminal and migration law is evidenced by the introduction of wider cancellation powers in 2014 and the creation of the Australian Border Force in 2015. The stated objectives of this shift are to protect national security and public safety by quickly and efficiently identifying and removing ‘risky’ non-citizens. Drawing on the experience of the United States, we have argued that the utility of this shift should be treated with some scepticism. US attempts to manage crime through migration have not resulted in an increase in removals of non-citizens for terrorism related offences. Rather, they have facilitated the removals of many thousands of non-citizens for low level crimes, many of whom were long-term residents. Stringent enforcement of removal laws has resulted in punitive outcomes for individuals and the separation of families.

The experience of the United States should act as a cautionary tale for Australia. We predict that, as a consequence of a move towards a risk management model, the new visa cancellation powers could have adverse punitive effects on long-term permanent residents in Australia, rather than ensuring national security. This calls for further research in the coming years to chart how the new cancellation powers are exercised, the characteristics of those being deported, and the extent to which mitigating factors against removal were taken into account by decision-makers. Such research will be important in determining whether this growing merger between criminal and migration law in Australia should be supported, or as others have suggested, whether it is a case of ‘using a sledgehammer to crack a nut’. In the case of the latter, we suggest that law reform measures will be necessary.

## Footnotes

1 For example, states that are a party to the 1951 UN Convention Relating to the Status of Refugees and its 1967 Protocol are obligated not to return (refoule) a person to frontiers where they may face persecution. The obligation of *non-refoulement* is contained in Article 33 of the Refugee Convention and is widely recognised as a fundamental principle of the Refugee Convention.

2 For example, Australian migration law permits visa cancellation on the basis that a visa-holder has not complied with a condition of their visa. Once a visa is cancelled, a person becomes an ‘unlawful non-citizen’ who is subject to detention and removal. See *Migration Act 1958* (Cth) ss 116 (cancellation power), s 189 (detention) and s 198 (removal).

3 Stumpf argues that crimmigration can be explained by membership theory: the idea that positive rights and privileges are only afforded to members (citizens) as part of the social contract between the government and its people. Those who are not members (i.e. non-citizens) are not party to the social contract and therefore have no claim to the rights held by members, including the right not to be removed. For Gibney, the exclusionary policies of states can be explained by the concept of ‘partialism’: the idea that ‘[s]tates have the right to distribute membership as they please because without this right people could not protect and reproduce their cultural identity’ (Gibney, 2004, p. 31). As such, exclusionary migration policies may be justified on the grounds of preserving cultural identity.

4 Table 60 shows that of the 633,918 people excluded from the United States between 1892 and 1984, only 14,287 were excluded due to a criminal or narcotics violation.

5 8 U.S.C § 1227(a)(2)(A)(iv).

6 8 U.S.C § 1227(a)(2)(A)(v).

7 8 U.S.C § 1227(a)(2)(C).

8 8 U.S.C § 1227(a)(2)(E).

9 8 U.S.C § 1227(a)(2)(A)(i)-(ii).

10 See Anti-Drug Abuse (ADA) Act of 1988, Pub. L. No. 100–690, 102 Stat. 4181, 4387, § 7342. The definition of ‘aggravated felony’ can be found in 8 U.S.C § 1101(a)(43).

11 AEDPA, Pub. L. No. 104–132, tit. IV, 110 Stat. 1214 (1996).

12 IIRIRA, Pub. L. No. 104–208, div. C, 110 Stat. 3009, 3009–546 (1996).

13 AEDPA § 440(e).

14 IIRIRA, Pub. L. No. 104–208, Div. C., § 321, 110 Stat. 3009–546, 3009–627 (1996) (codified as amended at 8 U.S.C. § 1101(a) (43) (F)-(G) (2000)).

15 See IIRIRA § 321(b); 8 U.S.C. § 1101(a)(43) (2000).

16 Padilla v. Kentucky, 130 S. Ct. 1486 (2010).

17 Padilla v. Kentucky, 130 S. Ct. 1473, 1478 (2010) (emphasis added).

18 According to ICE’s website, its mission is to protect ‘America from the cross-border crime and illegal immigration that threaten national security and public safety’. See https://www.ice.gov/overview.

19 According to its website, the US Customs and Border Protection is ‘one of the world’s largest law enforcement organisations and is charged with keeping terrorists and their weapons out of the U.S. while facilitating lawful international travel and trade’.

20 See INA, 8 U.S.C. § 1357 (g) authorizing the Attorney General to enter into agreements with states to deputize state officers and employees to perform the functions of immigration officers.

21
*Minister for Immigration and Multicultural and Indigenous Affairs v Nystrom* (2006) 228 CLR 566.

22 The concept of ‘jurisdictional error’ in Australian administrative law revolves around the question of whether the decision-maker has acted beyond the powers given to him or her by the legislation.

23 The next two paragraphs are drawn from a discussion of the US approach in K Hoang, *‘The rise of crimmigration in Australia: Importing laws and exporting lives’* in The Palgrave Handbook on Criminology and the Global South (forthcoming 2017).

24 The decision of the Federal Court was appealed to the High Court of Australia where the Minister’s cancellation decision was re-instated - *Minister for Immigration and Multicultural and Indigenous Affairs v Nystrom* (2006) 228 CLR 566. The High Court held that ‘The discretion to cancel a visa conferred upon the Minister under s 501(2) of the Act is unfettered in its terms’[126] noting that ‘Parliament has left it to the Minister to decide the matters which are relevant to whether a person who fails the character test should be permitted to remain in Australia. Considerations relevant to the exercise of the power depend on the nature, scope and purpose of the power, understood in its context in the Act.’ [128] (Heydon and Crennan JJ, with whom Gleeson CJ agreed).
